# Prevalence of and risk factors for HBV and HCV among incarcerated people who inject drugs in Iran: A cross sectional study

**DOI:** 10.1186/s12879-020-05541-2

**Published:** 2020-10-31

**Authors:** Ghobad Moradi, Parvin Mohamadi, Bushra Zareie, Mohammad Aziz Rasouli, Mohammad Mehdi Gouya, Saeede Jafari

**Affiliations:** 1 Social Determinants of Health Research Center, Research Institute for Health Development, Kurdistan University of Medical Sciences, Sanandaj, Iran; 2 Department of Medical Sciences, Sanandaj Branch, Islamic Azad University, Sanandaj, Iran; 3 Clinical Research Development Center, Kowsar Hospital, Kurdistan University of Medical Sciences, Sanandaj, Iran; 4 Iranian Center for Communicable Diseases Control, Ministry of Health and Medical Education, Tehran, Iran; 5 Student Research Committee, Faculty of Medicine, Kurdistan University of Medical Sciences, Pasdaran Ave, Sanandaj, Iran

**Keywords:** HCV, HBV, Iran, Prevalence, Prison, PWID

## Abstract

**Background:**

This study aimed at determining the prevalence of and risk factors for hepatitis B virus (HBV) and hepatitis C virus (HCV) among incarcerated people who inject drugs (PWID) in Iran in 2015–16.

**Methods:**

The required data was collected from a database provided by Iranian national bio-behavioral surveillance surveys (BBSSs) on 11,988 prisoners selected from among 55 prisons in 19 provinces in 2015–16. The data on demographics and behavioral variables were collected through interviews and the status of exposure to HBV and HCV were determined using ELISA blood test. A total of 1387 individuals with a history of drug injection in their lifetime were enrolled into the study. Data were analyzed using the survey package in Stata/SE software, Version 14.0. Univariate and multivariate logistic regression tests were used to investigate the relationships between risk factors and outcomes.

**Results:**

The mean age of the incarcerated PWID was 36.83 ± 8.13 years. Of all the studied subjects, 98.46% were male and 50.97% were married. The prevalence of HCV and HBV among the subjects were 40.52 and 2.46%, respectively. The prevalence of HCV was associated with age ≥ 30 years, being single, illiteracy and low level of education, prison term> 5 years, history of piercing, and extramarital sex in lifetime (*P* < 0.05).

**Conclusions:**

The prevalence of HCV is alarmingly high. In general, it is recommended to adopt measures to screen and treat patients with HCV and vaccinat incarcerated PWID without a history of vaccination against HBV.

**Supplementary Information:**

The online version contains supplementary material available at 10.1186/s12879-020-05541-2.

## Background

Viral hepatitis is currently one of the most important causes of mortality and morbidity worldwide [1]. Unlike the declining trend of the prevalence of human immunodeficiency virus, the burden of hepatitis is increasing worldwide and 96% of the deaths from hepatitis are related to two main types of viral hepatitis, namely hepatitis B virus (HBV) and hepatitis C virus (HCV) [2]. Although both of them are preventable and HCV is even treatable, the diseases caused by these viruses are considered as health challenges.

There are differences between HBV and HCV in terms of prevalence that are attributed to differences between their geographic distribution. HBV is more common in Africa and the Western Pacific Region while HCV is more common in the Eastern Mediterranean and European region. However, according to recent reports published by the World Health Organization (WHO), the prevalence of HBV and HCV worldwide in 2015 were 3.5 and 1%, respectively [2].

Studies have shown that a large number of cases with HBV and HCV are only observed in specific groups such as prisoners and people who inject drugs (PWID) [3], The reported prevalence of HBV and HCV among prisoners across the world are 4.8 and 15.1%, respectively [4]; in addition, the reported prevalence of HBV and HCV among PWID are 9.1 and 52.3%, respectively [5]. Iran is no exception and has a similar status; in other words, the prevalence of HBV and HCV are higher among prisoners and PWID than among the general population. As reported by other studies, the prevalence of HBV and HCV, respectively, are 2.5 and 9.5% among prisoners [6], 30.9 and 51.5%, among PWID [7], and 0.9% [7] and 0.6% among the general population [8]. It might be attributed to different reasons. First, a significant percentage of prisoners are incarcerated due to drug-related crimes [9], therefore, there is a large population of PWID in prisons. Secondly, due to the lack of interventions and proper care and treatment services, shared injection is a common practice in prisons [10] and it has been proved that non-sterile shared injections is one of the main routes of the transmission of blood-borne diseases (BBD) [11, 12]. Therefore, the prevalence of HBV and HCV among incarcerated PWID is expected to be significantly higher than the figures mentioned above.

Based on the results of Daneshmand and Davoodian’s study on incarcerated PWID in Iran, the duration of imprisonment is one of the factors associated with the prevalence of HBV and HCV [13, 14]. A population-based study on Iranian prisoners conducted by Moradi et al. showed that the prevalence of HBV and HCV were associated with a history of drug use; in addition, the prevalence of HCV was associated with a history of tattooing and age > 30 years old [6]. Silverman et al. conducted a study in Mexico and found that the prevalence of HCV in prisons was positively associated with injecting drug use, aging, frequency of incarcerations, and history of tattooing, while it was negatively associated with higher levels of education and being married [15].

The high prevalence of BBD in prisons can be considered as both a threat and an opportunity for the health system. It is a threat because the prison is not an environment isolated from the community and the increase in the prevalence of the diseases in prisons is associated with an increase in the burden of diseases in the community, especially after the release of prisoners. It is also an opportunity, the gathering of high-risk groups in a site i.e. in prisons, facilitates the access to infected and high-risk individuals. Moreover, it provides health policy-makers with a chance to design and implement appropriate behavioral surveys to accurately estimate the burden of diseases, identify related risk factors, tailor appropriate interventions, and effectively implement diagnostic, preventive, and therapeutic programs especially for eligible and vulnerable individuals [16]. It not only facilitates but also speeds up the achievement of the World Health Organization’s (WHO) goal set to eliminate HBV and HCV by 2030 [2]. The aim of this study was to determine the prevalence of and risk factors for HBV and HCV among Iranian incarcerated PWID using national bio-behavioral surveillance surveys (BBSSs) in 2015–2016.

## Methods

### Data source

The present cross-sectional study is part of a series of reiterating biobehavioral surveillance surveys (BBSSs) on HBV and HCV among Iranian prisoners that were carried out in 2015 and 2016. The methodology of BBSSs was the same in both rounds of the study and it is comprehensively presented in published BBSSs [6, 17], however, a summary is provided below. The study population included all Iranian prisoners who were in prison at the time of the study. Using multi-stage random sampling method, the subjects were selected. First, all the 31 provinces of Iran were categorized into three groups, namely the north, center, and south groups. Then, three provinces from each category, and 2–4 prisons from each province were randomly selected (9 provinces and 26 prisons in 2015 and 10 provinces and 29 prisons in 2016 were included in the study). Considering the average population of incarcerated people in each province and prison per year, the required number of samples in each prison was determined through probability-proportional-to-size sampling method. Finally using systematic random sampling, eligible prisoners (Iranian, age > 18 years, and with a history of prison term of at least 1 month at the time of the study) were recruited from the roster of all inmates in the prison; after obtaining a written informed consent, the selected people were enrolled into the study. Accordingly, over the two rounds, 12,800 Iranian prisoners including 6200 persons in 2015 and 6600 persons in 2016 that accounted for 0.06 and 0.10 of the total population of prisoners in the selected cities were recruited. Of the 12,800 selected subjects, 11,988 persons (5507 persons in 2015 and 6481 persons in 2016) participated in the surveys. Of these participants (11988), 1387 prisoners had a history of injecting drug use in their lifetime, all of whom formed our study population.

### Data collection tool

#### Demographic and behavioral data

In both rounds, a questionnaire-based interview was performed to collect data on demographics and behavioral status of the participants. The questionnaires, included seven sections (collecting data on demographic variables, criminal records, history of sexually transmitted diseases (STD), knowledge about HBV and HCV, and history of high-risk behaviors including drug abuse, tattooing and piercing). All demographic and behavioral data were collected via self-report. The variables were defined as below:

#### Illiteracy: inability to read and write

##### Primary education

This category included people who just attended a primary school. In Iran, primary school lasts 5 years. In other words, this category included people who had successfully passed at minimum of 1 and a maximum of 5 years of education in a primary school.

##### Junior high school

This category included people who attended a junior high school and no more. In Iran, junior high school lasts from 1 to 3 years. In other words, this category included people who had successfully passed in minimum of 1 and a maximum of 3 years in a junior high school.

##### Diploma

This category included people who had completed primary, junior and senior high school successfully. In other words, this category included people who had successfully completed at least 5 years of primary school, 3 years of junior high school, and 3 years of senior high school.

##### University education

It included people who had successfully finished high school education and were graduated in an academic filed in a university.

##### History of imprisonment

This category included people with a history of incarceration other than the present term of incarceration.

##### Number of incarcerations

Frequency of records of imprisonment other than the present term of incarceration; it was calculated for those with a previous history of imprisonment.

##### Prison term in the lifetime

Sum of the years of imprisonment; it was calculated for those with a history of imprisonment.

##### History of tattooing in the lifetime

History of injecting ink into the skin in the lifetime in every place.

##### History of tattooing in prison

History of injecting ink into the skin in prison; it was calculated for those with a history of tattooing in the lifetime.

##### History of piercing in the lifetime

History of making a hole in ear, lip, mouth, navel, and breast by a needle and cupping in the lifetime in every place.

##### History of piercing in prison

History of making a hole in ear, lip, mouth, navel, and breast by a needle and cupping at prison; it was calculated for those with a history of piercing in the lifetime.

##### History of extramarital sex in the lifetime

Engagement of a married person in a sexual affair with someone other than his/her legal spouse.

##### Number of sexual partners in the lifetime

Number of people, other than a legal spouse, that a married person had sexual activity with, either heterosexual or homosexual. It was calculated for those with a history of extramarital sex in the lifetime.

##### History of STD

Symptomatic self-reported history of wounds or abnormal secretion from genital area (abnormal in terms of amount, color, and odor), and STDs diagnosed by a physician.

#### Serologic data

Blood samples were collected and sent for serological testing to verify the exposure to HBV and HCV. Dried blood spot samples were collected from individuals who consented to participate in the study; the collected samples were stored under a standard condition. The presence of HBsAg and HCV antibodies were evaluated through assessing optical density (od) and cut-off points in ELISA (Enzyme-Linked Immunosorbent Assay) using Dia Pro kits (Diagnostic Bioprobes Srl, Italy). The validity of the results was verified using repeat testing for both positive and negative cases. All the tests on blood samples were carried out in the Pasteur Institute of Iran.

The questioning team in each prison consisted of two trained individuals: a) an interviewer selected among people working in health and treatment departments of prisons, b) a blood sampler selected among the staff working in a university of medical sciences.

### Statistical analysis

Data were analyzed using the survey package in Stata/SE software, Version 14.0. Following the survey design, weighting, clustering, and stratification were performed on the data collected from a total of 12,800 samples. Weights of the samples were determined using poststratification method, and postweight instrument. The frequency distribution of poststrata was determined through assessing people’s willingness to participate in the study. Finally the data required for this study was analyzed using descriptive statistics (frequency and mean) and analytical statistics (chi-square test, univariate and multivariate logistic regression). Since there were a large number of variables, only the variables with a *P* < 0.2 in the chi-square test were selected for multivariate analysis.

## Results

The mean age of the participants in this study was 36.83 ± 8.13 years. The study of demographic variables showed that the majority of the incarcerated PWID were male (98.46%), and half of them were married (50.91%) (Table 1). The prevalence of HCV and HBV among 1387 incarcerated PWID were 40.52% (CI95%: 34.22–47.15) and 2.46% (CI95%: 1.60–3.75), respectively.

**Table 1:** Prevalence of HCV and HBV among the incarcerated PWID

Variables	Total	HCV (***N*** = 589)		HBV (***N*** = 37)	
		N (%)^a^ (95% CI)	***P***-value	N (%)^a^ (95% CI)	***P***-value
**Gender**					
Female	12	3 (29.42) (7.41–68.46)	0.527	1 (4.29) (0.36–35.71)	0.598
Male	1375	586 (40.70) (34.44–47.27)		36 (2.43) (1.63–3.60)	
**Age (Year)**					
< 30	216	55 (24.25) (14.14–38.37)	0.004	6 (3.90) (1.72–8.61)	0.240
≥ 30	1168	532 (43.64) (37.45–50.04)		31 (2.18) (1.31–3.61)	
Missing	3				
**Marital Status**					
Single	587	274 (45.79) (37.07–54.77)	0.069	20 (3.12) (2.05–4.72)	0.459
Divorced/widowed	181	79 (40.08) (27.97–53.55)		4 (1.99) (0.59–6.51)	
Married	616	235 (36.48) (30.42–43.01)		13 (2.02) (0.95–4.27)	
Missing	3				
**Level of educational**					
Illiterate /primary	513	222 (41.26) (34.40–48.47)	0.002	16 (2.66) (1.31–5.35)	0.893
Junior high school / Diploma	819	354 (41.28) (35.15–47.68)		19 (2.22) (1.44–3.42)	
University education	53	11 (18.07) (8.85–33.37)		2 (4.22) (0.79–19.69)	
Missing	2				
**History of imprisonment**					
No	227	85 (37.69) (30.18–45.84)	0.393	5 (2.11) (0.54–7.84)	0.775
Yes	1153	502 (41.23) (34.15–48.69)		32 (2.55) (1.68–3.85)	
Missing	7				
**Number of incarcerations**					
1–2	453	161 (32.51) (24.92–41.14)	< 0.001	12 (2.41) (1.08–5.30)	0.264
3–4	341	159 (44.78) (36.46–53.40)		14 (3.90) (2.35–6.41)	
≥ 5	355	179 (48.24) (39.52–57.06)		6 (1.50) (0.49–4.53)	
Missing	4				
**Prison term in the lifetime**					
≤ 5	607	238 (36.51) (27.98–45.98)	< 0.001	16 (2.08) (1.13–3.80)	0.331
> 5	241	128 (52.77) (46.10–59.34)		8 (3.75) (1.42–9.50)	
Missing	305				
**History of tattooing in the lifetime**					
No	412	146 (35.70) (29.21–42.77)	0.013	11 (2.22) (1.26–3.87)	0.744
Yes	972	442 (42.71) (35.88–49.83)		25 (2.49) (1.48–4.18)	
Missing	3				
**History of tattooing in prison**					
No	532	241 (39.87) (32.36–47.89)	0.365	14 (2.63) (1.48–4.62)	0.827
Yes	433	196 (45.08) (35.41–55.14)		11 (2.40) (1.10–5.18)	
Missing	7				
**History of piercing in the lifetime**					
No	681	259 (36.16) (29.48–43.42)	0.005	12 (1.56) (0.67–3.55)	0.635
Yes	700	329 (45.17) (38.36–52.18)		25 (3.39) (2.40–4.78)	
Missing	6				
**History of piercing in prison**					
No	582	274 (44.45) (37.80–51.31)	0.846	22 (3.70) (2.44–5.58)	0.262
Yes	109	50 (45.70) (33.32–58.64)		2 (1.60) (0.43–5.83)	
Missing	9				
**History of extramarital sex in the lifetime**					
No	332	123 (32.31) (23.97–41.94)	0.070	7 (1.81) (0.60–5.36)	0.440
Yes	944	414 (43.10) (34.95–51.64)		26 (2.76) (1.81–4.18)	
Missing	111				
**Number of heterosexual/ homosexual partners in the lifetime**					
1	112	43 (34.17) (25.61–43.89)	0.216	4 (3.22) (1.05–9.47)	0.216
2–3	448	216 (45.98) (34.45–57.96)		14 (2.96) (1.71–5.08)	
> 3	295	114 (37.06) (25.78–49.95)		3 (0.95) (0.41–2.19)	
Missing	89				
**Condom use in extramarital sex**					
Never	204	85 (39.04) (27.25–52.28)	0.325	5 (1.88) (0.74–4.71)	0.277
Sometime	519	257 (42.81) (34.17–51.92)		19 (3.24) (1.98–5.26)	
Always	139	68 (48.39) (37.85–59.07)		2 (1.39) (0.34–5.50)	
Missing	10				
**History of STD** ^b^ **in the last year**					
No	1275	538 (40.09) (34.20–46.28)	0.193	34 (2.47) (1.57–3.84)	0.983
Yes	110	50 (47.97) (33.94–62.32)		3 (2.43) (0.78–7.37)	
Missing	2				

^a^ weighted prevalence, ^b^ Sexually transmitted diseases

The results of chi-square test showed that the prevalence of HCV was significantly higher in individuals with 30 years of age and older (as compared with people under 30 years of age), illiterate prisoners and those with an education level lower than university education (as compared with those with an university education), prisoners with a higher frequency and length of imprisonment (as compared with those with a lower frequency and length of imprisonment), subjects with a history of tattooing (as compared with those without a history of tattooing in the lifetime), and those with a history of piercing in the lifetime (as compared with those without a history of piercing). However, there was no significant difference between the mentioned subgroups of prisoners and the prevalence of HBV (Table 1).

To achieve a proper multivariate model, a total of eight variables with *P* < 0.2 in chi-square test were entered into the model (the variables included age, marital and educational status, prison term, tattooing, piercing, and extramarital sex in the lifetime, and history of STD). The results of multivariate logistic regression indicated that HCV exposure had a statistically significant relationship with being 30 years and older (adjusted OR (AOR): 3.50, 95% CI: 1.75–6.99), being single (AOR: 1.72, 95% CI: 1.13–2.62), non-university education (AOR: 3.42, 95% CI: 1.50–7.81), history of imprisonment for more than 5 years (AOR: 1.65, 95% CI: 1.27–2.14), history of piercing in the lifetime (AOR: 1.53, 95% CI: 1.15–2.04), and history of extramarital sex (AOR: 1.61, 95% CI: 1.03–2.50) (Table 2).

**Table 2:** Multivariate logistic regression analysis of factors associated with the prevalence of HCV among incarcerated PWID

Variables	OR (95% CI)	***P***-value	AOR (95% CI)	***P***-value
**Age (Year)**				
< 30	1		1	
≥ 30	2.42 (1.33–4.39)	0.005	3.50 (1.75–6.99)	0.001
**Marital Status**				
Married	1		1	
Divorced/ widowed	1.16 (0.71–1.90)	0.532	1.30 (0.65–2.58)	0.440
Single	1.47 (1.04–2.07)	0.029	1.72 (1.13–2.62)	0.012
**Level of education**				
University education	1		1	
Junior high school – Diploma	3.19 (1.50–6.77)	0.004	3.53 (1.51–8.26)	0.005
Illiterate – primary	3.18 (1.49–6.81)	0.004	3.42 (1.50–7.81)	0.005
**Prison term in the lifetime (year)** ^a^				
≤ 5	1		1	
> 5	1.94 (1.48–2.55)	< 0.001	1.65 (1.27–2.14)	< 0.001
**History of tattooing in the lifetime**				
No	1		1	
Yes	1.34 (1.07–1.69)	0.014	0.94 (0.68–1.32)	0.730
**History of piercing in the lifetime**				
No	1		1	
Yes	1.45 (1.13–1.87)	0.005	1.53 (1.15–2.04)	0.005
**History of extramarital sex in the lifetime**				
No	1		1	
Yes	1.59 (0.96–2.63)	0.071	1.61 (1.03–2.50)	0.036
**History of STD**^b^ **in the last year**				
No	1		1	
Yes	1.38 (0.84–2.25)	0.194	1.81 (0.93–3.52)	0.079

^a^ The variables of the number of incarceration (OR:1.39, CI_95%_: 1.20–1.62) and the prison term (OR:1.94, CI_95%_: 1.48–2.55) had a correlation with each other. The variable of the prison term was entered into multivariate logistic regression model because of its higher significant OR

^b^ Sexually transmitted diseases

## Discussion

This study aimed at identifying risk factors for HBV and HCV among one of the most important high-risk groups, i.e. incarcerated PWID in Iran. Comparing the prevalence of HCV in our study with the prevalence of the disease reported by other studies in 2015, it was found that the ratio of the prevalence of HCV among incarcerated PWID to general population was 67.53 (40.52% vs 0.6%) [8], to prisoners was 4.3 (40.52% vs 9.48%) [6], and to non-incarcerated PWID was 0.79 (40.52% vs 51.46%) [7]. In addition, comparing the prevalence of HBV in our study with the prevalence of the disease reported by other studies, it was found that the ratio of the prevalence of HBV among incarcerated PWID to the above mentioned subgroups in the same year was 2.73 (2.46% vs 0.90%) [7], 0.99 (2.46% vs 2.48%) [6], and 0.08 (2.46% vs 30.90%) [7]. The facts stated earlier in the introduction can justify the higher prevalence of this disease among the incarcerated PWID than among prisoners and general population. However, the prevalence of the disease among this group was lower than that among non-incarcerated PWID, which may be attributed to the fact that although injections are practiced in prisons, it is a prohibited action; therefore, the frequency of injection, and in particular the frequency of the practice of shared and non-sterile injection are lower in prisons than in the community. Furthermore, prisoners are prone to hide their real injection status. However, based on the findings of a study by Falla et al. in Europe, the prevalence of HBV and HCV in prisoners is higher than that among injecting drug users [3]. It highlights the fact that imprisonment results in the gathering of a large group of patients and provides a chance to implement health-related interventions, including diagnosis, treatment, and prevention programs.

The results of simultaneous investigation of the prevalence of HBV and HCV in this study and other studies in Iran show that the prevalence of HCV is significantly higher than the prevalence of HBV among prisoners and drug users [6, 7], while among the general population, HBV is slightly more prevalent than HCV [7]. Hence, when resources and facilities are limited, it is recommended to give priority to HCV than HBV. In addition, in order to achieve the goal of eliminating hepatitis in a community, it is necessary to allocate a large part of investment and health interventions to manage and control HCV.

Based on the results of this study, the prevalence of HCV among incarcerated PWID was 40.52%; although it is a notable figure, it is significantly lower than the figures reported for this group of people in other studies [18]. The reported rate at the global level is 64% [19]. Moreover, according to Behzadifar et al., its rate in Iran is 53%. The observed difference may be attributed to differences between the types of two studies. Behzadifar et al.’s study was conducted as a meta-analysis investigating papers published from 2004 to 2016, and the highest prevalence rate was observed in years prior to 2010 [20].

As the most important findings of this study, it was found that the prevalence of HCV among incarcerated PWID was associated with an age of 30 years and older, being single, illiteracy and low level of education, more than 5 years of imprisonment, history of piercing in the lifetime, and history of extramarital sex. The association between HCV prevalence and age and lengthy imprisonment may be due to the fact that people aged 30 years and older and those with longer imprisonment terms are more likely than people younger than 30 years and those with shorter imprisonment terms to have prolonged contact with high-risk groups. Hence, they are more prone to be exposed to the risk factors. The relationship between HCV and being single can be attributed to the fact that married people have a commitment and adherence to ethics that is less observed in single persons [21]. The observed relationship between illiteracy and low level of education and HCV may also be attributed to the lack of information and knowledge or the lack of social prestige in illiterate people and those with a low level of education; it is worth noting that university education has a restraining effect on socially negative behaviors. Piercing was also identified as a risk factor, which might be attributed to shared needle injection and incorrect sterilization of piercing tools [22]. In addition, high-risk sexual activity is also among the most proved routes of STD transmission. Some of the risk factors identified in this study are infrequently reported in a limited number of studies conducted on incarcerated PWID. Silverman-Retana et al.’s study in Mexico showed that the prevalence of HCV was positively associated with older ages (age over 40) and the frequency of imprisonment, while it had a negative relationship with high level of education and being married [15]. In a study by Dolan et al. in Australia, it was found that history of imprisonment and high level of education were associated with the prevalence of HCV among incarcerated PWID [23]. Davoodian et al.’s study in Iran showed that the prevalence of HCV was associated with the length of incarceration [14].

Based on the results of chi-square test, there was no *P*-value less than 0.2 when assessing the relationship between HBV and independent variables. Therefore, multivariate logistic regression analysis was not performed to assess the prevalence of HBV (Table 2) and there was no risk factor for the prevalence of HBV among the incarcerated PWID. It may be attributed to the low prevalence of HBV infection among the studied people. This finding is consistent with the results of Davoodian et al.’s study [14]; however, the results of Daneshmand’s study in Iran indicated an association between HBV and length of imprisonment [13]. This controversy may be attributed to differences between the settings of the two studies. Daneshmand’s study was conducted among incarcerated PWID in just one province (Isfahan) out of a total of 31 provinces of Iran, and the prevalence of HBV in the mentioned province was significantly higher (27.2%), while the prevalence reported in this study (2.46%) was achieved through conducting surveys in 19 provinces.

This study was part of BBSSs on HBV and HCV in prisons in Iran, and the required data was collected through self-reports. Therefore, information bias, including recall bias and social desirability bias were the most important limitations of the study; in order to reduce their effects, complementary questions were used in each section to identify unreal answers to the main questions.

## Conclusion

Prior to this study, there was no report on the prevalence of and risk factors for HBV and HCV among Iranian incarcerated PWID at the national level. Since drug injection and imprisonment are two important risk factors for HCV, it is important to pay special attention to this group of prisoners to significantly reduce the prevalence of HBV and HCV. The results of this study showed that the prevalence of HBV and HCV among incarcerated PWID is alarmingly high. In general, it is recommended to implement HCV screening and treatment programs and perform HBV vaccination plans for incarcerated PWID without a history of vaccination. In case of the lack or shortage of resources and facilities in the health care system, in order to reduce HCV prevalence more effectively, it is suggested to implement screening and treatment programs through prioritizing incarcerated PWID with short terms (who will be soon released back into the community) and those who practice high-risk behaviors such as tattooing, piercing, and extramarital sex.

## Supplementary Information

**Additional file 1.**

## Data Availability

The datasets used and/or analyzed during the current study can be made available by the corresponding author on a reasonable request.

## Abbreviations

HBV: Hepatitis B virus
HCV: Hepatitis C virus
PWID: People who inject drugs
WHO: World Health Organization
BBD: Blood-borne diseases
STD: Sexually transmitted diseases

## References

[1] Stanaway JD, Flaxman AD, Naghavi M, Fitzmaurice C, Vos T, Abubakar I (2016). The global burden of viral hepatitis from 1990 to 2013: findings from the global burden of disease study 2013. Lancet.

[2] World Health Organization (2017). Global hepatitis report 2017: World Health Organization.

[3] Falla AM, Hofstraat SHI, Duffell E, Hahné SJM, Tavoschi L, Veldhuijzen IK (2018). Hepatitis B/C in the countries of the EU/EEA: a systematic review of the prevalence among at-risk groups. BMC Infect Dis.

[4] Dolan K, Wirtz AL, Moazen B, Ndeffo-mbah M, Galvani A, Kinner SA (2016). Global burden of HIV, viral hepatitis, and tuberculosis in prisoners and detainees. Lancet.

[5] Degenhardt L, Peacock A, Colledge S, Leung J, Grebely J, Vickerman P (2017). Global prevalence of injecting drug use and sociodemographic characteristics and prevalence of HIV, HBV, and HCV in people who inject drugs: a multistage systematic review. Lancet Glob Health.

[6] Moradi G, Gouya MM, Azimizan Zavareh F, Mohamadi Bolbanabad A, Darvishi S, Aghasadeghi MR (2018). Prevalence and risk factors for HBV and HCV in prisoners in Iran: a national bio-behavioural surveillance survey in 2015. Tropical Med Int Health.

[7] Amiri FB, Mostafavi E, Mirzazadeh A (2016). HIV, HBV and HCV coinfection prevalence in Iran-a systematic review and meta-analysis. PLoS One.

[8] Mirminachi B, Mohammadi Z, Merat S, Neishabouri A, Sharifi A H, et al. Update on the Prevalence of Hepatitis C Virus Infection Among Iranian General Population: a Systematic Review and Meta-Analysis. Hepat Mon. 2017;17(2):e42291. 10.5812/hepatmon.42291.

[9] Carpentier C, Royuela L, Montanari L, Davis P (2017). The global epidemiology of drug use in prison. Drug Use in Prisoners: Epidemiology, Implications, and Policy Responses.

[10] Cunningham EB, Hajarizadeh B, Amin J, Bretana N, Dore GJ, Degenhardt L (2018). Longitudinal injecting risk behaviours among people with a history of injecting drug use in an Australian prison setting: the HITS-p study. Int J Drug Policy.

[11] Bretaña NA, Boelen L, Bull R, Teutsch S, White PA, Lloyd AR (2015). Transmission of hepatitis C virus among prisoners, Australia, 2005–2012. Emerg Infect Dis.

[12] Hajarizadeh B, Grebely J, Dore GJ (2013). Epidemiology and natural history of HCV infection. Nat Rev Gastroenterol Hepatol.

[13] Daneshmand D, Nokhodian Z, Adibi P, Ataei B (2013). Risk prison and hepatitis B virus infection among inmates with history of drug injection in Isfahan, Iran. Scientific World Journal.

[14] Davoodian P, Dadvand H, Mahoori K, Amoozandeh A, Salavati A (2009). Prevalence of selected sexually and blood-borne infections in injecting drug abuser inmates of Bandar Abbas and roodan correction facilities, Iran, 2002. Braz J Infect Dis.

[15] Silverman-Retana O, Serván-Mori E, McCoy SI, Larney S, Bautista-Arredondo S (2017). Hepatitis C antibody prevalence among Mexico City prisoners injecting legal and illegal substances. Drug Alcohol Depend.

[16] Stone J, Martin NK, Hickman M, Hutchinson SJ, Aspinall E, Taylor A (2017). Modelling the impact of incarceration and prison-based hepatitis C virus (HCV) treatment on HCV transmission among people who inject drugs in Scotland. Addiction..

[17] Moradi G, Jafari S, Zarei B, Mahboobi M, Azimian Zavareh F, et al. Prevalence and Risk Factors for Hepatitis B and Hepatitis C Exposure in Iranian Prisoners: a National Study in 2016. Hepat Mon. 2019;19(7):e91129. 10.5812/hepatmon.91129.

[18] Snow KJ, Young JT, Preen DB, Lennox NG, Kinner SA (2014). Incidence and correlates of hepatitis C virus infection in a large cohort of prisoners who have injected drugs. BMC Public Health.

[19] Larney S, Kopinski H, Beckwith CG, Zaller ND, Jarlais DD, Hagan H (2013). Incidence and prevalence of hepatitis C in prisons and other closed settings: results of a systematic review and meta-analysis. Hepatology..

[20] Behzadifar M, Gorji HA, Rezapour A, Bragazzi NL (2018). Prevalence of hepatitis C virus infection among prisoners in Iran: a systematic review and meta-analysis. Harm Reduct J.

[21] Kerr L, Kendall C, Guimarães MDC, Mota RS, Veras MA, Dourado I (2018). HIV prevalence among men who have sex with men in Brazil: results of the 2nd national survey using respondent-driven sampling. Medicine.

[22] Ravlija J, Vasilj I, Marijanović I, Vasilj M (2014). Risk behaviour of prison inmates in relation to HIV/STI. Psychiatr Danub.

[23] Dolan K, Teutsch S, Scheuer N, Levy M, Rawlinson W, Kaldor J (2010). Incidence and risk for acute hepatitis C infection during imprisonment in Australia. Eur J Epidemiol.

