# Supplementary material for: Prevalence of and risk factors for HBV and HCV among incarcerated people who inject drugs in Iran: A cross sectional study
Source: BMC Infect Dis. 2020 Oct 31;20:806. doi: 10.1186/s12879-020-05541-2 (PMC7603667; doi:10.1186/s12879-020-05541-2)
Supplement: Supplementary file 1 — Additional file 1. [file 12879_2020_5541_MOESM1_ESM.docx]

Consent form

| The present questionnaire was designed to assess the prevalence of hepatitis B and C in Iranian prisons. The results of this study will be used to plan for the provision of services in the country's prisons. In this interview, you will not be asked for your name, nor your address, and your answers will be kept strictly confidential. We will only use the sum of the participants' answers for statistical analysis. Private questions may also be asked during this interview. It is to be noted that although your cooperation in honestly answering all the questions is very valuable to us, you are free to answer any question that does not seem appropriate to you. If you want to participate in this review, please sign this form.  Interviewer's name:  Date of interview: ….….. - …… - ……  Participant sing: |
| --- |

Is the person willing to participate in the hepatitis B and C surveillance survey? Yes€ No

If yes, complete the questionnaire.

| **Questionnaire code** | **Interviewer code** | **Prison Code** | **Province Code** | **Year of surveillance survey** |
| --- | --- | --- | --- | --- |
|  |  |  |  |  |

| **Date of blood sampling** | **Date of interview** | **Interviewer's name** | **Blood sampler's name** |
| --- | --- | --- | --- |
|  |  |  |  |

| **Part 1: Demographic characteristics** | |
| --- | --- |
| **Question** | **Answer** |
| 1.Gender | Male €  Female€ |
| 2. On what date were you born?  (Record as Year – Month- Day) | ….….. - …… - …… |
| 3. What is your educational level? | Illiterate €  Reading and Writing €  Primary €  Junior high school €  High School €  Diploma €  University education €  I do not know / I do not remember €  No answer € |
| 4. What was your job in the year before current incarceration? | Employee €  Retired €  Soldier €  Farmer €  Free €  Jobless €  Salesman €  Students €  Housewives €  Other (job should be mentioned.) …….  I do not know / I do not remember €  No answer € |
| 5. How is your marital status now? | Single (never married) €  Married €  Divorced €  Widowed €  I do not know / I do not remember €  No answer € |

| **Part 2: History of imprisonment** | |
| --- | --- |
| **Question** | **Answer** |
| 6. Since when have you been imprisoned?  (Record as Year – Month- Day) | ….….. - …… - …… |
| 7. Have you ever had a history of imprisonment in the last ten years?  (If yes, ask the next two questions, otherwise go to part 3) | Yes€  No  I do not know / I do not remember €  No answer € |
| 8. How many times have you been imprisoned in the last ten years? | ….. times  I do not know / I do not remember €  No answer € |
| 9. How long have you been in prison in your previous imprisonment? | …… day/days  …… month/ months  …… Year/years |

| **Part 3: History of drug use** | |
| --- | --- |
| **Question** | **Answer** |
| 10. Have you ever consumed illegal drugs?  (If yes, ask the following questions, otherwise go to part 4) | Yes€  No  I do not know / I do not remember €  No answer € |
| 11. How old were you the first time you used illegal drugs? | ….. years  I do not know / I do not remember €  No answer € |
| 12. What drugs have you ever used? | Opium/ opium extract €  Ecstasy €  Heroin €  Temgesic/ Norgesic €  Crack €  Cocaine €  Methamphetamine €  Sedatives drugs €  Illicit methadone €  Other (drugs should be mentioned.) …….  I do not know / I do not remember €  No answer € |
| 13. How did you consume the drug at the first time? | Inhalation €  Oral €  Pipes €  Injection €  Other (methods should be mentioned.) …  I do not know / I do not remember €  No answer € |
| 14. What has been the most common method of drug use? | Inhalation €  Oral €  Pipes €  Injection €  Other (methods should be mentioned.) …  I do not know / I do not remember €  No answer € |
| 15. How did you consume drug the last time? | Inhalation €  Oral €  Pipes €  Injection €  Other (methods should be mentioned.) …  I do not know / I do not remember €  No answer € |
| 16. Where did you use the drug at the first time? | Subjects home €  Park €  Prison €  Friends home €  Other (place should be mentioned.) …  I do not know / I do not remember €  No answer € |
| 17. What was the most common drug you used before current imprisonment?  (Guidance: The most common, should be one answer) | Opium/ opium extract €  Ecstasy €  Heroin €  Temgesic/ Norgesic €  Crack €  Cocaine €  Methamphetamine €  Sedatives drugs €  Illicit methadone €  Other (drugs should be mentioned.) …….  I do not know / I do not remember €  No answer € |
| 18. Do you have a history of drug injection?  (If yes, ask the questions from19 to 25, otherwise inquiry question 26) | Yes€  No  I do not know / I do not remember €  No answer € |
| 19. How old were you the first time you injected drug? | ….. years  I do not know / I do not remember €  No answer € |
| 20. Where did you inject the drug at the first time? | Subjects home €  Park €  Prison €  Friends home €  Other (place should be mentioned.) …  I do not know / I do not remember €  No answer € |
| 21. What drug did you inject the last time?  (Guidance: If she/he had injected two drugs or more simultaneously, multiple choices can be choosed)) | Opium/ opium extract €  Ecstasy €  Heroin €  Temgesic/ Norgesic €  Crack €  Cocaine €  Methamphetamine €  Sedatives drugs €  Illicit methadone €  Other (drugs should be mentioned.) …….  I do not know / I do not remember €  No answer € |
| 22. Do you have a history of shared injection (injection with a syringe or needle used by others)? | Yes€  No  I do not know / I do not remember €  No answer € |
| 23. Where did you almost get the used syringes? | It was myself €  I took it from someone €  I found €  I do not know / I do not remember €  No answer € |
| 24. Have you ever lent or sold your used syringe to someone for using? | Yes€  No  I do not know / I do not remember €  No answer € |
| 25. Do you have a history of shared injection (injection with a syringe or needle used by others) in prison? | Yes€  No  I do not know / I do not remember €  No answer € |
| 26. Are you under treatment with Methadone maintenance therapy (MMT) or buprenorphine? | Yes€  No  I do not know / I do not remember €  No answer € |

| **Part 4: History of tattooing** | |
| --- | --- |
| **Question** | **Answer** |
| 27. Have you ever tattooed?  (If yes, ask the next three questions, otherwise go to part 5) | Yes€  No  I do not know / I do not remember €  No answer € |
| 28. When was the last time you got a tattoo?  (Record as Year – Month- Day) | ….….. - …… - ……  I do not know / I do not remember €  No answer € |
| 29. Where was the last time you got a tattoo? | Inside prison €  Outside prison €  I do not know / I do not remember €  No answer € |
| 30. Who did the tattoo for you? | Barbers €  Peddlers €  Friends €  Inmates €  Subjects themselves €  Family members €  I do not know / I do not remember €  No answer € |

| **Part 5: History of piercing** | |
| --- | --- |
| **Question** | **Answer** |
| 31. Have you ever had body piercing including cupping or each pierce in ear, nose, mouth, tongue, navel and breast?  (If yes, ask the next three questions, otherwise go to part 6) | Yes€  No  I do not know / I do not remember €  No answer € |
| 32. When was the last time you got a pierce?  (Record as Year – Month- Day) | ….….. - …… - ……  I do not know / I do not remember €  No answer € |
| 33. Where was the last time you got a pierce? | Inside prison €  Outside prison €  I do not know / I do not remember €  No answer € |
| 34. Who did the pierce for you? | Physician €  Barbers €  Peddlers €  Friends €  Inmates €  Subjects themselves €  Family members €  I do not know / I do not remember €  No answer € |

| **Part 6: History of sexual relationship** | |
| --- | --- |
| **Question** | **Answer** |
| 35. Have you ever had a sexual relationship?  (If yes, ask the following questions, otherwise go to part 7) | Yes€  No  I do not know / I do not remember €  No answer € |
| 36. How old were you the first time you had sex? | ….. years  I do not know / I do not remember €  No answer € |
| 37. When was the last time you had sex? | …… day/days ago  …… month/ months ago  …… Year/years ago  I do not know / I do not remember €  No answer € |
| 38. Have you had sex since current imprisonment?  (If yes, ask questions 39, otherwise inquiry questions 40) | Yes€  No  I do not know / I do not remember €  No answer € |
| 39. When was this sexual relationship? | Conjugal rooms in prisons €  During recess time (temporarily leave) €  I do not know / I do not remember €  No answer € |
| 40. Have you ever had sex with someone other than your spouse (extramarital sex)?  (If yes, ask following questions, otherwise go part7) | Yes€  No  I do not know / I do not remember €  No answer € |
| 41. How many people have you had sex with other than your spouse? | ….. person/ persons  I do not know / I do not remember €  No answer € |
| 42. How many nonmonetary partners have you had sex with that sex have not been in exchange for money or drugs? | ….. person/ persons  I do not know / I do not remember €  No answer € |
| 43. How many temporary partners have you had sex with that sex have been in exchange for money or drugs? | ….. person/ persons  I do not know / I do not remember €  No answer € |
| 44. How you had sex? | Oral sex €  Vaginal sex €  Anal sex €  I do not know / I do not remember €  No answer € |
| 45. Have you ever had sex with someone who had one of the diseases and you were aware of his/her disease?  (Guidance: multiple choices can be choosed) | Hepatitis B virus (HBV) €  Hepatitis C virus (HCV) €  Human immunodeficiency virus (HIV/AIDS) €  Sexually transmitted diseases (STD) €  No  I do not know / I do not remember €  No answer € |
| 46. Did you use a condom during extramarital sex? | Always €  Sometimes €  Rarely €  Never €  I do not know / I do not remember €  No answer € |
| 47. Have you ever had sex with a man or boy (male gender)?  (Guidance: This question should only be asked of males) | Yes, in recent 6 months€  Yes, in last year €  Yes, before last year€  No  I do not know / I do not remember €  No answer € |

| **Part 7: History of sexually transmitted diseases** | |
| --- | --- |
| **Question** | **Answer** |
| **In the recent year of current incarceration** | |
| 48. Have you had a genital sore or ulcer in the recent year? | Yes €  No   I do not know / I do not remember €  No answer € |
| 49. Have you had an unusual genital discharge (in terms of amount color odor) in the recent year? | Yes €  No   I do not know / I do not remember €  No answer € |
| 50. Where have you referred to for treatment of genital sore/ulcer and discharge in the recent year?  (Guidance: multiple choices can be choosed) | Traditional medicine center (Herbal remedies) €  Clinic/ triangular clinic €  Pharmacy €  Self-medication €  Other (place should be mentioned.) …….  No-treatment action € |
| **In the year before current incarceration** | |
| 51. Have you had a genital sore or ulcer in the last year? | Yes €  No   I do not know / I do not remember €  No answer € |
| 52. Have you had an unusual genital discharge (in terms of amount color odor) in the last year? | Yes €  No   I do not know / I do not remember €  No answer € |
| 53. Where have you referred to for treatment of genital sore/ulcer and discharge in the last year?  (Guidance: multiple choices can be choosed) | Traditional medicine center (Herbal remedies) €  Clinic/ triangular clinic €  Pharmacy €  Self-medication €  Other (place should be mentioned.) …….  No-treatment action € |

| **Part 8: History of HBV & HCV** | |
| --- | --- |
| **Question** | **Answer** |
| 54. Were you born to a mother with hepatitis B or C? | Yes €  No   I do not know / I do not remember €  No answer € |
| 55. Have you ever lived with someone who had hepatitis? | Yes €  No   I do not know / I do not remember €  No answer € |
| 56. Have you ever had a blood transfusion? | Yes €  No   I do not know / I do not remember €  No answer € |
| 57. Have you received the Hepatitis B vaccine?  (If yes, ask questions 58, otherwise go part 9) | Yes €  No   I do not know / I do not remember €  No answer € |
| 58. How many times have you got HBV vaccination? | One €  Two €  Three and more € |

| **Part 9: Knowledge about the transmission of HBV & HCV** | |
| --- | --- |
| **Question** | **Answer** |
| 59. Have you ever heard of hepatitis B and C?  (If yes, ask following questions, otherwise go part 10) | Yes €  No   I do not know / I do not remember €  No answer € |
| 60. Can hepatitis B and C be transmitted from parents to children? | Yes €  No   I do not know / I do not remember €  No answer € |
| 61. Is it possible a healthy looking person be infected with the hepatitis virus? | Yes €  No   I do not know / I do not remember €  No answer € |
| 62. Can hepatitis B and C be transmitted through sexual activity with infected person? | Yes €  No   I do not know / I do not remember €  No answer € |
| 63. Can hepatitis B and C be transmitted through shaking hands, hugging, kissing, sitting next to infected person, and other casual contact? | Yes €  No   I do not know / I do not remember €  No answer € |
| 64. Can hepatitis B and C be transmitted through tattooing, cupping, piercing, medical and dental services in unsafe places and with contaminated and non-sterile equipment? | Yes €  No   I do not know / I do not remember €  No answer € |
| 65. Can hepatitis be transmitted while sharing needles used to drug injection | Yes €  No   I do not know / I do not remember €  No answer € |
| 66. Is the statement that "the main route of transmitting hepatitis C in our country is to use shared syringes/needles" correct? | Yes €  No   I do not know / I do not remember €  No answer € |
| 67. Can both hepatitis B and C be cured easily? | Yes €  No   I do not know / I do not remember €  No answer € |
| 68. Is there a hepatitis B vaccine? | Yes €  No   I do not know / I do not remember €  No answer € |
| 69. Is there a hepatitis C vaccine? | Yes €  No   I do not know / I do not remember €  No answer € |

| **Part 10: History of testing for HBV & HCV** | |
| --- | --- |
| **Question** | **Answer** |
| 70. Have you ever been tested for hepatitis B and C?  (If yes, ask the following questions, otherwise inquiry question 73 ) | Yes €  No   I do not know / I do not remember €  No answer € |
| 71. When was the last time you tested for hepatitis? | …… day/days ago  …… month/ months ago  …… Year/years ago  I do not know / I do not remember €  No answer € |
| 72. Where have the last time you referred to for test of hepatitis? | Behavioral diseases counseling center €  Clinic or laboratory €  At the time of my arrest €  Blood donation center €  Drop-in center €  Triangular clinic/ prison clinic €  Other (place should be mentioned.) ……. |
| 73. Are you willing to be tested for hepatitis B and C? | Yes €  No  |
| 74. Date of hepatitis B test  (Record as Year – Month- Day) | ….….. - …… - …… |
| 75. Date of hepatitis C test  (Record as Year – Month- Day) | ….….. - …… - …… |
| 76. Test result  for hepatitis B virus infection | Positive €  Negative € |
| 77. Test result  for hepatitis C virus infection | Positive €  Negative € |
